# Extending the psychological support services to the underage family members of critically ill patients: the ariadne program

**DOI:** 10.1186/2197-425X-3-S1-A647

**Published:** 2015-10-01

**Authors:** E Demetriadou, M Kokkinou, E Epiphaniou, G Christodoulou, N Stylianides, T Kyprianou, ARIADNE Study Group

**Affiliations:** Nicosia General Hospital, Intensive Care, Nicosia, Cyprus; Intensive Care Forum (NGO), Nicosia, Cyprus; Open University of Cyprus, Nicosia, Cyprus; St Georges Univ. of London Medical Program at the Univ. of Nicosia, Nicosia, Cyprus

## Introduction

Hospitalization in the Intensive Care Unit (ICU) can have negative bio-psychological effects for the patients but also for their families; There is paucity in literature, though, regarding the psychological support of underage family members, having limited access to the ICU. Mobile health (mHealth) applications have been extensively used in health care services, however, there is limited data regarding their use in facilitating psychological support.

## Objectives

**a)** To design and develop a State of the Art web based platform to facilitate the accessibility of the underage family members (aged 4-18) of ICU patients to psychological support services

**b)** to integrate an assessment and intervention protocol and **c)** to assess its feasibility, accessibility, applicability and performance.

## Methods

**a) The platform** was developed through a multidisciplinary team and integrates state of the art web technologies (Single Page Application-Web Sockets-Web-RTC), patient health records, two way audio-visual comm. and a digital palette of intervention protocol tools.

**b) The development of an assessment and intervention protocol**

Psychological and tele-psychological assessment and intervention protocol was developed using the Delphi consensus methodology: expert psychologists - semi-structured interviews - questionnaires - consensus meeting - final assessment and intervention protocol.

**c) The platform assessment:** First phase (May 2014 - April 2015): Children are supported with In Vivo psychological sessions. Second phase (April - September 2015), mHealth platform will be used instead. The applicability, participation, perceived quality of services, performance (Likert scale), number of sessions required according to pre-specified criteria and other indices will then be compared Three questionnaires to children / parents will be used prior and post interventions.

## Results

Here we are presenting:

- The final design and structure of the platform as well its pilot use phase,
- Delphi consensus process results,
- final battery of assessment tools (mental state, quality of the service), as well as personalised interventions based on subject's needs.

During the 1st phase, 700 patients were admitted in the ICU, 360 stayed more than 48 hrs (51.5 %), mortality rate 15.1%, SMR 0.98). Following recruitment methodology (piloted from May and intensified with consecutive cases from November 2014), 91 sessions (In Vivo) were conducted in 48 children and adolescents (mean age 10 years) of 24 ICU patients. Also 25 family counselling sessions were conducted involving 19 guardians.

## Conclusions

This is the first -so far known- existing study, extending ICU psychological and tele-psychological services, designed to specifically involve minors. mHealth technologies could impact and guide the way children are managed while having a loved one in the ICU.

**ARIADNE program** is co-funded by EEA grants (http://eeagrants.org/project-portal/project/CY03-0016) and the Intensive Care Forum (NGO)

